# Section 1Tunable broadband terahertz absorbers based on multiple layers of graphene ribbons

**DOI:** 10.1038/s41598-017-16220-9

**Published:** 2017-11-20

**Authors:** Dingbo Chen, Junbo Yang, Jingjing Zhang, Jie Huang, Zhaojian Zhang

**Affiliations:** 1 Center of Material Science, National University of Defense Technology, Changsha, 410073 China; 2 State Key Laboratory on Advanced Optical Communication Systems and Networks, Peking University, Beijing, 100871 China

## Abstract

A novel metamaterial structure consisting of multiple graphene/dielectric layers and metallic substrate is proposed to achieve the broadband absorption response at terahertz (THz) frequencies. Utilizing the phase modulation effect generated by graphene ribbons, the bright-dark field is formed to suppress the reflection based on interference theory in a wide period. By irregularly stacking four graphene ribbons of varying widths on four dielectric layers with unequal thickness in a period, we merge successive absorption peaks into a broadband absorption spectrum successfully. The absorption decreases with fluctuations as the incident angle increases. The position of the absorption spectrum can be dynamically tuned by a small change in the Fermi level of graphene instead of re-optimizing and re-fabricating the device. In addition, the bandwidth of the absorber can be further improved by means of increasing the graphene/dielectric layers. The structure proposed in this paper has potential applications in tunable terahertz photonic devices such as dynamic broadband filters, modulators and sensors.

## Introduction

Metamaterials (MMs) are a kind of artificial structural materials with exotic properties not easily obtained or completely unavailable in nature^1–4^. Over the past decades, the rapid developments of MMs have attracted extensive attention from material scientists, physicists and engineers, because various types of fascinating metamaterial devices had been theoretically and experimentally investigated^5–7^. The appearance of split ring resonators^8^, cut wire pairs^9^ and metallic ribbons^10^ have greatly enriched the MMs theory^11,12^. Various types of exotic features that are unavailable in nature have been theoretically and experimentally investigated. MMs perfect absorber^13^, which is an important branch of MMs, has attracted a lot of attention currently, because the inevitable losses in metallic plasmonic nanostructures can be used for this research area. The first experimental demonstration of MMs absorber was given by Landy *et al*. in 2008^14^, in which the electric and magnetic fields of the incident are absorbed by two MMs resonators integrated on the top and the bottom sides of a substrate. Several studies presented designs for broadband absorbers in the terahertz and microwave regions by merging successive absorption peaks with different geometrical parameters resonators in a single unit cell^15–17^. So far, most of the MMs absorbers are realized at a fixed operating frequency. Once the devices are designed and fabricated, tuning the absorption window is very difficult to achieve^18^.

Graphene, a monolayer of carbon atoms arranged in a honeycomb lattice, is the first truly 2-D (two-dimensional) material to be observed in nature^19^, acting as a prospective, alternative candidate for plasmonics at mid-infrared and THz frequencies^20–22^. Because of the fantastic electromagnetic properties, provided by the unique electronic band structure that the energy-momentum relation for electrons is liner over a wide range of energies^23,24^, graphene has attracted extensive attention in optics, electricity and magnetism. More importantly, one of the remarkable properties of graphene is that its Fermi level can be dynamically changed by means of external gate voltages^25^, which makes graphene an ideal material for realizing optical tunable devices. Currently, a variety of graphene-based MMs have been devised for enhancing optical absorption, such as, graphene ribbons^26^, discs^27^, even double-layer graphene structures^28^. However, the narrow absorption spectrum of the majority of graphene-based absorbers has greatly restricted its development.

In this paper, we propose an absorber, based on multilayer graphene ribbons, to suppress the reflectivity of incidence terahertz light in a broadband spectral range. A series of successive absorption peaks are merged into a broadband absorption spectrum, by irregularly stacking four graphene/dielectric layers on an optically thick golden substrate. This absorber is simulated and optimized using Finite Element Method. Simulation results exhibits that the broadband absorption with general efficiency exceeding 95% (from 9.9 to 10.9 THz) and the full bandwidth at half-maximum (FWHM) of 1.6 THz has been achieved, on the condition of vertical incidence. Meanwhile, this absorber exhibits good absorption stability over a wide angle range of incidence from −30° to +30° at least. The absorption windows of such absorbers can be dynamically tuned by changing the Fermi level of graphene. The absorbers are composed of simple ribbons instead of complex structure as usual^29–34^. Furthermore, the bandwidth of the absorber can be further improved by increasing the graphene/dielectric layers, which is incredible for previous researches^35,36^.

## Results

**Figure 1:**
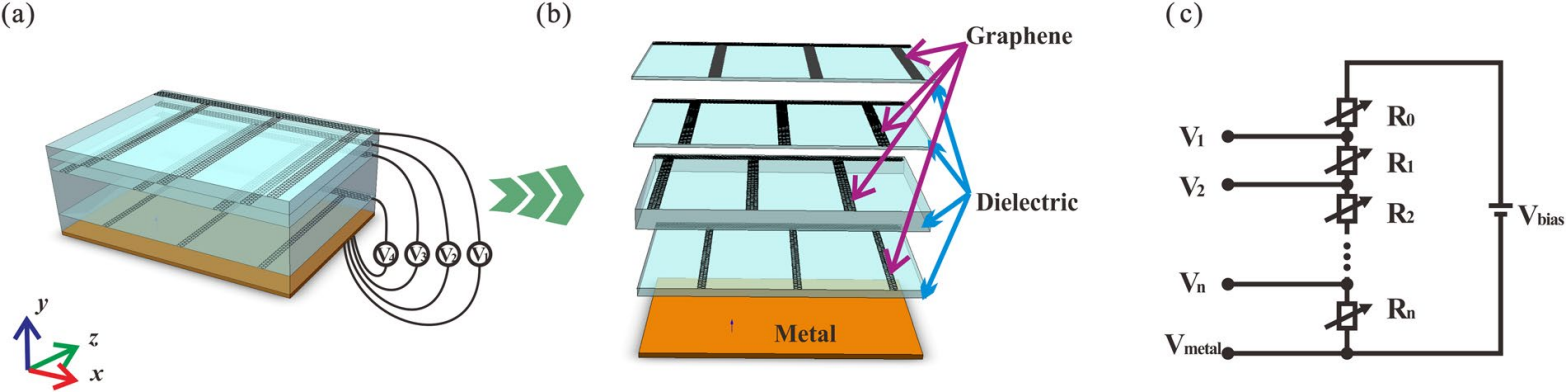
(**a**) Schematic diagram of the graphene-based broadband absorber, which consist of multiple subwavelength patterned graphene/dielectric layers stacking on a metal substrate. (**b**) An enlarged view of the unit cell on each layer. (**c**) The schematic of the external bias circuit. The branches of the voltage (*V*_1_~*V*_*n*_) are connected to different graphene layers respectively, and *V*_*metal*_ is connected to the metal reflector.

The proposed absorber composed of multiple graphene/dielectric layers stacking on an optically thick metal film is depicted schematically in Fig. 1. Figure 1(a) shows the structure diagram, and Fig. 1(b) shows le graphene/dielectric layers stacking on an optically thick metal film is depicted the enlarged view of the unit cell on each layer. In this structure, each layer of graphene combined with dielectric and the metallic substrate can be modeled as an asymmetric Fabry-Perot resonator, in which the graphene layer as a partially reflecting mirror in the front and a metallic fully reflecting mirror in the back. The Fermi level of each graphene layer can be tuned independently by varying the gate voltage. The gate voltage is provided by the external bias circuit as shown in Fig. 1(c). We can tune the Fermi levels of all the graphene ribbons uniformly or non-uniformly as assumed in the simulation by adjusting the resistors (*R*_1_~*R*_*n*_), which avoid the electrical isolation caused by insulator. The incident light is transverse magnetic (TM) polarization light.

### Single layer absorber

**Figure 2:**
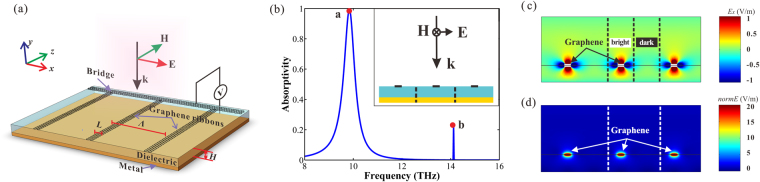
(**a**) Schematic of the single layer structure. (**b**) The simulation absorption spectrum for vertical incident light, and the illustration is the cross section of the single layer absorber. (**c**) *E*_*x*_ field component and (**d**) field intensity distributions of the reflected light at frequency of *f*_*a*_ = 9.84 THz (peak *a*).

In order to express the principle of reflection suppression more clarity, we take a simple single graphene/dielectric layer structure as example (Fig. 2(a)). The period *Λ* is fixed at 17 μm, the thickness of the dielectric *H* is 7 μm, the width of the graphene ribbon *L* is 1.7 μm and the Fermi level *E*_*f*_ is 0.64 eV. There is a bridge connect all the ribbons together, which makes the tuning of the Fermi level easier. In Fig. 2(b), the simulated absorption spectrum is shown and a narrow absorption peak (peak *a*) appears distinctly with the resonant frequency of *f*_*a*_ = 9.84 THz and the absorptivity is nearly 100%. The FWHM of the absorption peak is 0.6 THz (from 9.5 to 10.1 THz). The extra peak (peak *b*) in Fig. 2(b) is considered as split peak (SP) deriving from Rabi splitting which is the case of many atoms in an optical cavity^37,38^. At the position of absorption peak, the *E*_*x*_ field component distribution of the reflected light is shown in Fig. 2(c) and the electric field intensity (*normE*) distribution is shown in Fig. 2(d). It is obvious that the phase of reflected light in bright field area is different from that of dark field area. In essence, the phase of reflected light is modulated (modulation value is *π*) by the region contain graphene ribbons^39^, generating the phase matching between the bright and the dark field area. Therefore, the reflected light is suppressed due to the destructive interference of the bright field and dark field.

**Figure 3:**
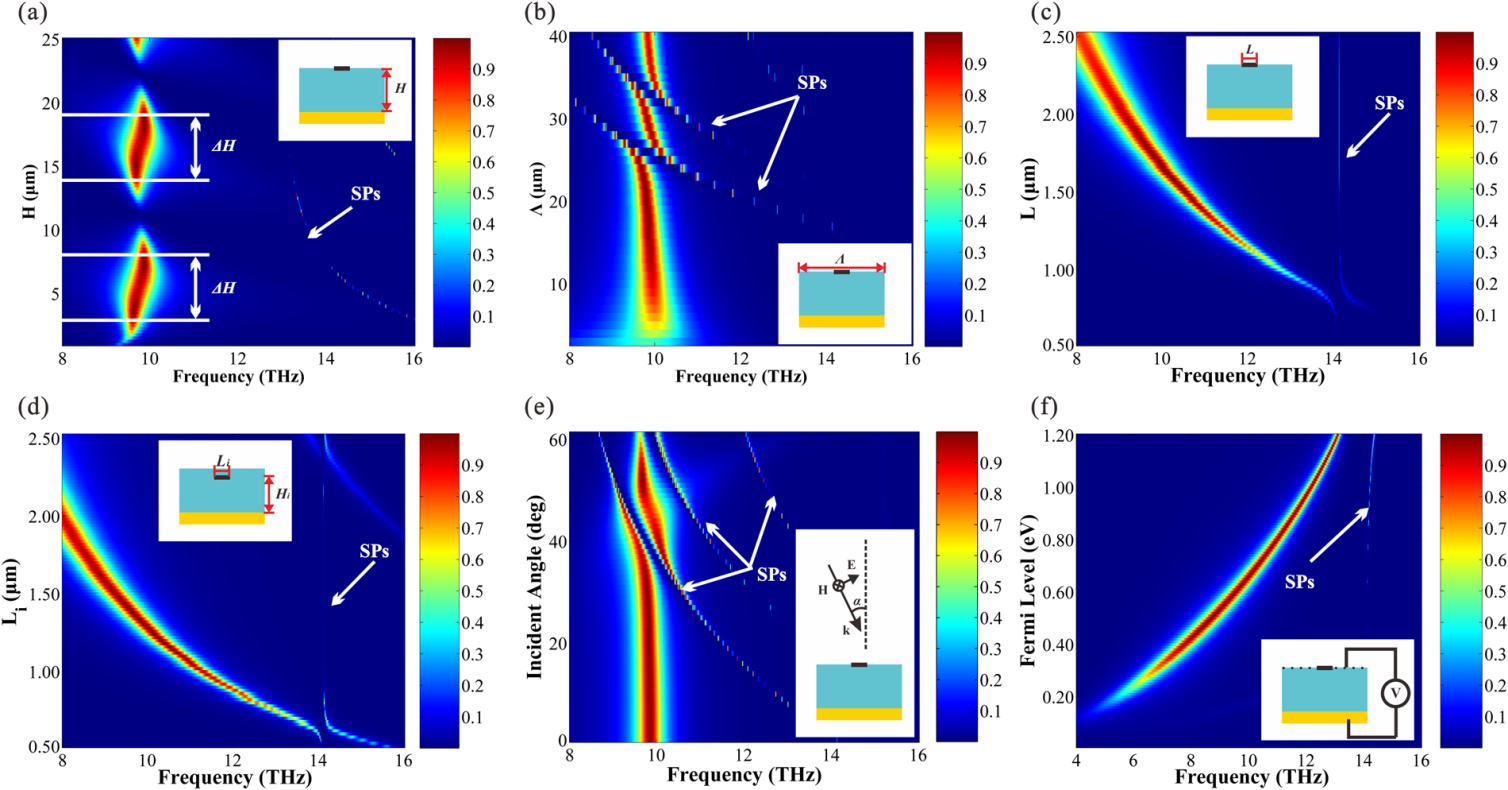
(**a**) Absorption spectra as a function of the thickness of the dielectric *H*. (**b**) Absorption spectra as a function of the period of the system *Λ*. (**c**) Absorption spectra as a function of the width of the ribbons (*L*), when the graphene is located in the interface of air and dielectric. (**d**) Absorption spectra as a function of the width of the ribbons (*L*_*i*_), when the graphene is located in inside of the dielectric. (**e**) Absorption spectra as a function of the incident angle. (**f**) Absorption spectra as a function of the Fermi level. The color bars represent the value of absorption.

To comprehend the characteristics of the system, we research the influence of absorption effects caused by the parameters variation based on the structure in Fig. 2(a). In the following, we analyze the parameters in sequence. Figure 3(a) exhibits the absorption spectra as a function of the thickness of the dielectric (*H*). If we change the dielectric thickness, the absorption shows periodic oscillations, and the oscillation period is about 11.5μm. The absorptivity of the system nearly reaches 100% within the scope of *ΔH* in each oscillation cycle. We define the area of *ΔH* as Efficient Absorption Range, and the value of *ΔH* is 5.5 μm (from 2.5 μm to 8μm in the first oscillation cycle, and from 14μm to 19.5μm in the second one). In the structure we proposed, *H* is fixed in the Efficient Absorption Range to achieve excellent absorption. Meanwhile, it is shown in Fig. 3(a) that the SPs move toward lower frequency (red-shift) with the increase of *H*.

From Fig. 3(b), one can clearly see that the absorption spectra as a function of the period (*Λ*). When *Λ* < 15 μm, the absorptivity increases with the increase of *Λ*. When *Λ* >21 μm, the SPs disturb the absorption spectra forming multiple Rabi splitting analogues. When *Λ* varies between 15 and 21 μm, the absorptivity keeps high because the equal intensity of bright field and dark field. In this work, the period of each system is fixed at 17 μm.

The response frequency of absorption peak is related to the width of the ribbons. In Figs. 3(c) and (d), we show the absorption spectra as a function of the width of graphene ribbons. The period is 17 μm and the dielectric thickness is 7 μm in both of the structures. The difference between the two structures is that, the graphene is located in the surface (Fig. 3(c)) and inside of the dielectric (Fig. 3(d)) respectively. The distance between the graphene and the metal reflector (*H*_*i*_) is 5.8 μm in Fig. 3(d). It is indicated that, as the width of graphene ribbons (*L* and *L*_*i*_) increase, the resonance frequency is pushed toward the direction of the low frequency (red shift). As is well known, the frequency of the absorption peak is associated with the permittivity of the media around the graphene film. Therefore, when *L* is equal to *L*_*i*_, the corresponding frequency of the absorption peak in Fig. 3(c) is larger than that in Fig. 3(d). The frequency of the SPs is almost constant, indicating that the SPs is not relevant to the width of the graphene ribbons.

It should be pointed out that incident light often irradiate with an oblique incidence angle in actual application. In order to investigate the absorption sensitivity to the oblique incident light, we vary the incident angle α from 0° to 60°. Figure 3(e) shows the absorption as a function of incident angle and frequency. The absorption peaks are stable in high efficiency when the incident angle varies between 0° and 30°. As the incident angle increase beyond 30°, the SPs move toward to lower frequency and disturb the absorption spectra forming Rabi splitting analogues.

For the sake of investigating the absorption response of the system on the Fermi level of the graphene, we present the absorption spectra of the system as a function of Fermi level in Fig. 3(f). We can obtain that, the position of absorption resonance peaks vary in a wide range as the tuning of *E*_*f*_, and the absorption effect is pretty good. The absorption peaks tend to exhibit blue shift with the increase of Fermi level, and there is slight effect on the SPs from Fermi level.

### Double layers absorber

**Figure 4:**
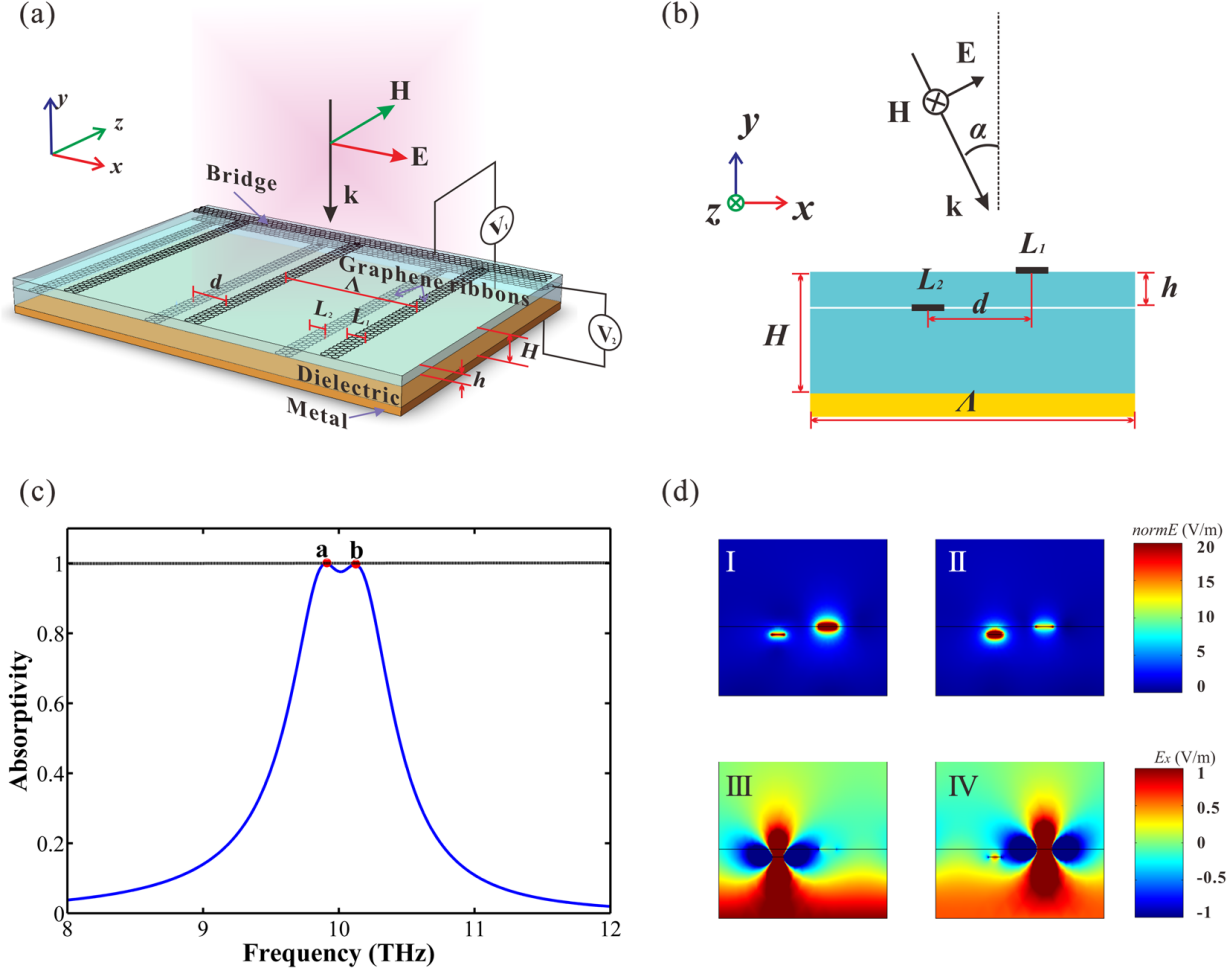
(**a**) Schematic of the double layers structure. (**b**) Schematic of the *x-y* section. *Λ* is the period of the absorber. *H* is the thickness of the dielectric. The width of the ribbons is *L*_1_ and *L*_2_ respectively. The distance between the two graphene ribbons is *d* and *h* in *x* direction and *y* direction respectively. Two graphene layers are tuned by different gate voltage. (**c**) The simulated absorption spectrum of the double layers absorber. There are two absorption peaks, *a* (9.90 THz) and *b* (10.12 THz). (**d**) I and III are the field intensity and *Ex* field component distributions of the reflected light at peak *a*; II and IV are the field intensity and *Ex* field component distributions of the reflected light at peak *b*.

Through the research of the single layer absorber, we have comprehended the basic principle of reflection suppression. The absorber has excellent absorption effect and tenability. However, the narrow absorption bandwidth limits the application of the absorber seriously. Therefore, expanding the bandwidth has been our primary goal. A feasible method to expand the bandwidth of the absorption spectra is that increasing the number of the graphene ribbons. Then, we put two graphene ribbons in each period instead of one. The schematic of the double layers absorber is shown in Fig. 4(a) and (b). As is shown, the period of the absorber is *Λ*, the thickness of the dielectric is *H*, the width of the graphene ribbons is *L*_1_ and *L*_2,_ the distance between the two graphene ribbons is *d* and *h* in *x* direction and *y* direction respectively. Two graphene layers are tuned by different gate voltage independently, the Fermi level of the top layer graphene is *E*_*f*1_ and the other one is *E*_*f*2_.

The parameters of the double layers absorber are set as follows: *Λ* is 17 μm, *H* is 7 μm, *L*_1_ is 1.7 μm, *L*_2_ is 1.23 μm, *d* is 5 μm, *h* is 0.8 μm, *E*_*f*1_ and *E*_*f*2_ are both 0.64 eV. The absorption spectrum is shown in Fig. 4(c). There are two successive absorption peaks (*a*, *f*_*a*_ = 9.90 THz; *b*, *f*_*b*_ = 10.12 THz) on the curve expanding the absorption spectrum. Figure 4(d) shows the field distribution of the *x-y* section at peak *a* and peak *b*. I and III are the field intensity and *E*_*x*_ field component distributions of the reflected light at peak *a*; II and IV are the field intensity and *E*_*x*_ field component distributions of the reflected light at peak *b*. We can obtain from Fig. 4(c) and (d) is that two ribbons correspond to different peak separately and that the bright and dark field is caused by different ribbons generating phase matching at corresponding absorption peak.

**Figure 5:**
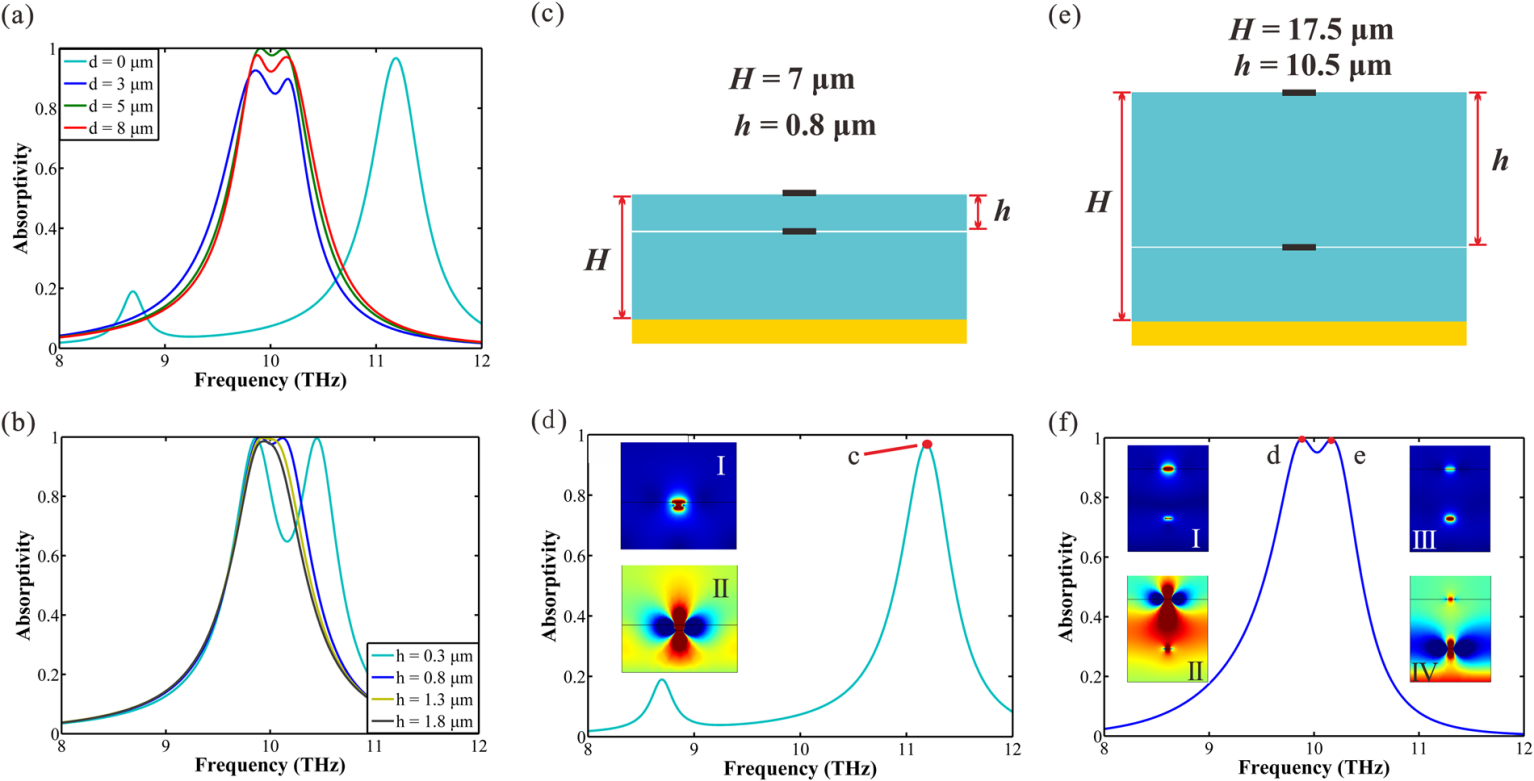
The simulated absorption spectra of the double layers absorber for varying the distance between the two graphene ribbons in (**a**) x direction and (**b**) *y* direction respectively. **(c**) Schematic of the double layers structure when *H* = 7 μm, *h* = 0.8 μm, *L*_1_ = 1.7 μm, *L*_2_ = 1.23 μm. (**d**) is the simulated absorption spectrum, the illustrations are the field intensity and *E*_*x*_ field component distributions of the reflected light at peak *c* (11.18 THz). (**e**) Schematic of the double layers structure when *H* = 17.5 μm, *h* = 10.5 μm, *L*_1_ = 1.7 μm, *L*_2_ = 1.2 μm. (**f**) is the simulated absorption spectrum, I and II are the field intensity and *E*_*x*_ field component distributions of the reflected light at peak *d* (9.88 THz), III and IV are that at peak *e* (10.16 THz).

The absorption effect of the broadband absorber depends on various factors, including the distance between the two graphene ribbons in *x* direction and *y* direction, the width of the graphene ribbons, the Fermi level and the incidence angle. Firstly, the distance between the two ribbons is taken into consideration. The simulated absorption spectra with varying distance between the two ribbons in *x* direction (*d*) and *y* direction (*h*) are plotted in Fig. 5(a) and (b), respectively, indicating that the absorption spectra vary with the change of the position of the ribbons. From Fig. 5(a), there is limited influence on the spectra as *h* varying from 0.8 to 1.8 μm. When *h* = 0.3 μm, the absorption curve has changed a lot, for the reason that the graphene below is close to the interface of the dielectric and air, varying the medium condition around the graphene ribbon and inducing mismatching of the phase matching condition. It can be obtained from Fig. 5(b) that, when *d* is 5 μm, the absorption effect is the best. The absorptivity decline as *d* increasing or decreasing, and the intensity of the two peaks become unbalanced. However, when *d* reduce to 0 μm, great changes have taken place in the curve of the absorption spectrum and new peaks have replaced the original peaks.

As shown in Fig. 5(c), the transverse distance *d* is 0 μm, the vertical distance *h* is 0.8 μm, the thickness of the dielectric is 7 μm, the width of the ribbons are 1.7 μm (*L*_*1*_) and 1.23 μm (*L*_2_) respectively. New absorption peaks are produced, because the ribbons are so close that form a new consortium. It can be confirmed in Fig. 5(d) that a new absorption peak *c* (11.18 THz) is formed and the two ribbons are as a consortium in the field distribution at peak *c*. The other small peak in the curve is the responding of high order mode. But, when the vertical distance *h* increases, the responding is changing. As shown in Fig. 5(e), *H* is 17.5 μm, *h* is 10.5 μm, *L*_*1*_ is 1.7 μm and *L*_2_ is 1.2 μm, two ribbons are in different Efficient Absorption Range. Here, *L*_*2*_ is slightly tuned for better broaden effect. The absorption spectra is shown in Fig. 5(f), there are two successive absorption peaks *d* (*f*_*d*_ = 9.88 THz) and *e* (*f*_*e*_ = 10.16 THz). The illustrations in Fig. 5(f) are the field intensity and *E*_*x*_ field component distributions of the two peaks respectively. The two peaks correspond to different ribbon, and there is no interaction between two ribbons. We can obtain that the bandwidth of the absorption spectrum can be further broaden by increasing graphene layers in different Efficient Absorption Range.

**Figure 6:**
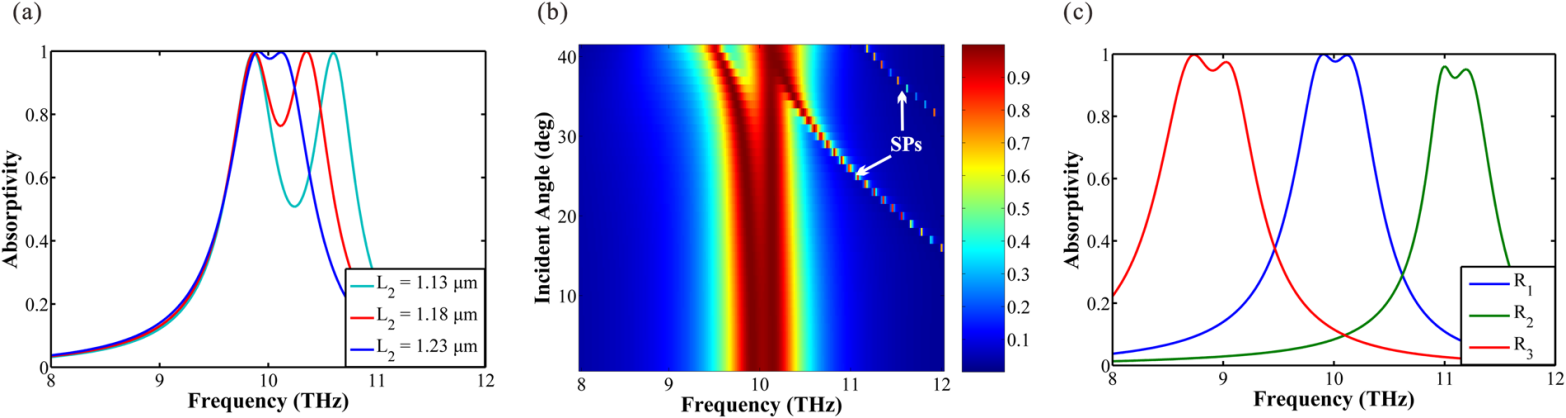
(**a**) The simulated absorption spectra of the double layers absorber for varying width of the lower ribbons (*L*_2_), the width of the upper graphene (*L*_1_) is fixed. (**b**) Absorption spectra of the double layers absorber as a function of incident angle. The color bar represents the value of absorption. (**c**) Absorption spectra of the double layers absorber as a function of different Fermi level (*E*_*f*_). The Fermi level of graphene ribbons can be tuned independently, the top layer graphene is *E*_*f*1_ and the other one is *E*_*f2*_. R_1_: *E*_*f1*_ = 0.64 eV, *E*_*f2*_ = 0.64 eV; R_2_: *E*_*f1*_ = 0.80 eV, *E*_*f2*_ = 0.78 eV; R_3_: *E*_*f1*_ = 0.50 eV, *E*_*f2*_ = 0.51 eV.

The simulated absorption spectra of the double layers absorber with varying width of the lower ribbon (*L*_*2*_) are plotted in Fig. 6(a). Here, the width of the upper layer ribbon (*L*_1_) is fixed and the other parameters are the same with that in Fig. 4(c). The absorption peaks of the corresponding ribbons tend to exhibit blue shift with the ribbon width decrease, and the intensity of the two peaks can be kept balance under present conditions. It can be seen that there is a good influence on the absorption if the distance of the two ribbons in *x* and *y* direction properly confirmed.

Figure 6(b) exhibits the absorption spectra, affected by the incident angle, and the other simulation parameters are same with that in Fig. 4(c). The absorption peaks are stable in high efficiency when the incident angle varies between 0° and 30°. As the incident angle increase beyond 30°, the SPs move toward to lower frequency and disturb the absorption spectra forming Rabi splitting analogues. Overall, the absorption exceeds 90% for incidence angles from 0° to 30° in which steep edge of the absorption bandwidth can be clearly observed.

The absorption spectra as a function of the Fermi level of the graphene ribbons are shown in Fig. 6(c). All the parameters except Fermi level are same with that in Fig. 4(c). The Fermi level of the top layer graphene is *E*_*f*1_ and the other one is *E*_*f2*_. In order to optimize the absorption spectra, the Fermi level can be adjusted independently. There are three absorption spectra (R_1_, R_2_ and R_3_) in Fig. 6(c). The Fermi level of the three curves are: R_1_, *E*_*f*1_ = *E*_*f*2_ = 0.64 eV; R_2_, *E*_*f1*_ = 0.80 eV, *E*_*f*2_ = 0.78 eV; R_3_, *E*_*f1*_ = 0.50 eV, *E*_*f2*_ = 0.51 eV. Both the absorptivity and the bandwidth of the absorption spectrum should be taken into account, when we adjust the Fermi level. As we can see, the absorption window shift toward higher frequency (blue-shift) with the increase of Fermi level, and the bandwidth of the absorption spectrum narrow down. The absorption window shift reversely (red-shift) with the decrease of the Fermi level, and the bandwidth of the spectrum become broader. However, in the considered range of the Fermi level, the absorption is always lager than 90% which is the powerful proof of the efficient tenability of the double layer absorber we proposed.

### Multiple layers broadband absorber

Based on the foundational theory above, the broadband absorber proposed broaden the absorption spectrum by stacking multiple graphene/dielectric layers and merging successive narrow absorption spectrum into a broad one. We can learn from Fig. 5(e) and (f) that the bandwidth of the absorber can be broaden unlimitedly as long as the added graphene layers are properly located in Efficient Absorption Range. Here, we take four layers absorber as an example to discuss the characteristics of the multiple layers absorber.

**Figure 7:**
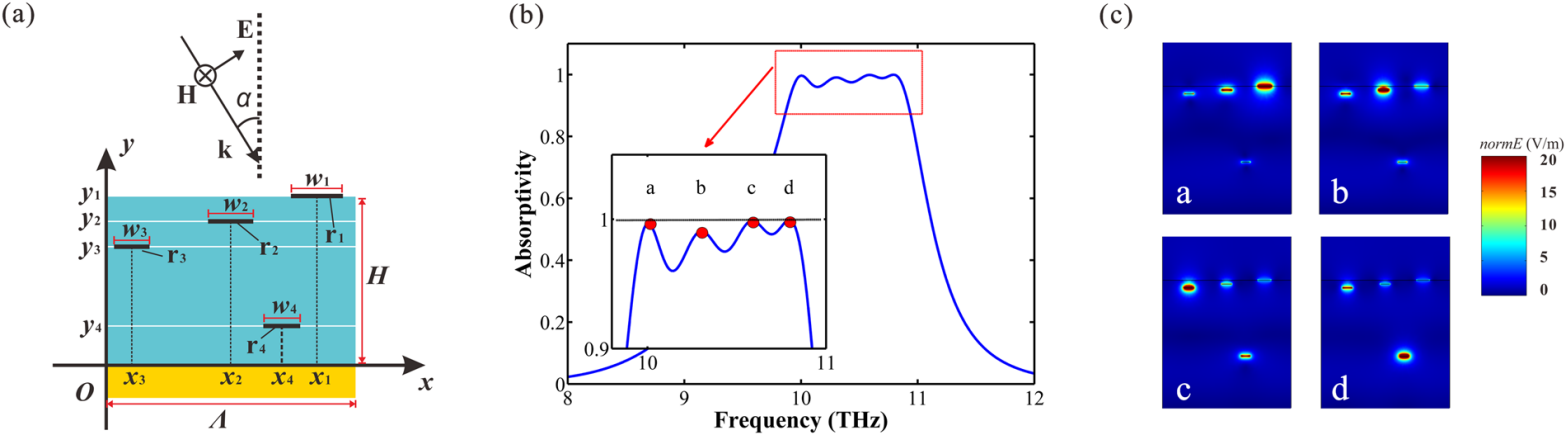
(**a**) Cross section of one period of the multiple layers absorber, where an *x-o-y* coordinates is set to provide convenience for accurate expression. (**b**) The simulated absorption spectrum of the broadband absorber. (**c**) The insets illustrates the field distribution at each absorption peak: *f*_*a*_ = 10.00 THz, *f*_*b*_ = 10.30 THz, *f*_*c*_ = 10.59 THz, *f*_*d*_ = 10.79 THz.

The schematic of the multiple layers absorber is shown in Fig. 1 and Fig. 7(a). Figure 7(a) shows the cross section of a period of the absorber. An *x-o-y* coordinates is set up, offering great help for accurate expression. The period of the ribbons is *Λ*. The total thickness of all the dielectric layers is fixed as *H*. There are four ribbons, named as *r*_*n*_ (*n* = 1, 2, 3, 4) respectively, distributing in each cycle. The width of ribbon *r*_*n*_ is assumed as *w*_*n*_. We assume that the coordinates of ribbon’s center is (*x*_*n*_, *y*_*n*_). The Fermi level of each ribbon is *E*_*fn*_.

The parameters of the absorber in our simulations are as follows. The period of the absorber *Λ* is 17 μm, the thickness of the dielectric *H* is 17 μm. The width and center coordinates of each ribbon are: ribbon *r*_*1*_, *w*_*1*_ = 1.680 μm, (13.5 μm, 17 μm); ribbon *r*_*2*_, *w*_*2*_ = 1.235 μm, (8.5 μm, 16.4 μm); ribbon *r*_3_, *w*_*3*_ = 1.130 μm, (3.5 μm, 16.0 μm); ribbon *r*_4_, *w*_*4*_ = 1.065 μm, (11.0 μm, 7.0 μm). The absorption simulation result is shown in Fig. 7(b), which plots the absorption spectrum of the device. It can be clearly seen that the absorption goes beyond 95% from 9.9 THz to 10.9 THz, covering a bandwidth of 1.0 THz, and the FWHM reaches 1.6 THz (from 9.5 THz to 11.1 THz). The resonance corresponds to different graphene ribbons in each of the absorption peak position, as depicted in Fig. 7(c). The insets a, b, c, d in Fig. 7(c) exhibit the field distribution of the absorption peaks *a* (*f*_*a*_ = 10.00 THz), *b* (*f*_*b*_ = 10.30 THz), *c* (*f*_*c*_ = 10.59 THz), *d* (*f*_*d*_ = 10.79 THz) respectively. It is significant in Fig. 7(c) that the absorption peaks *a*, *b*, *c*, *d* correspond to *r*_1_, *r*_2_, *r*_3_, *r*_4_, respectively.

**Figure 8:**
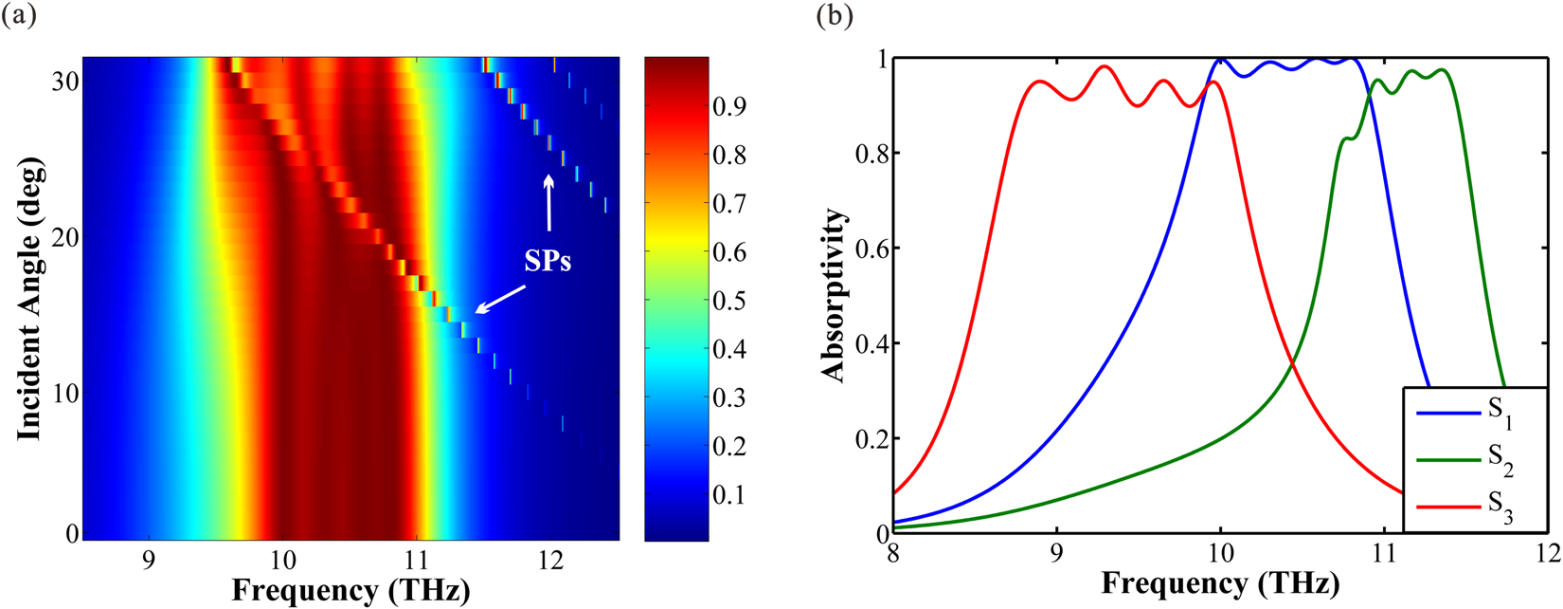
(**a**) Absorption spectra as a function of incident angle. The color bar represents the value of absorption. (**b**) Absorption spectra as a function of different Fermi level (*E*_*f*_). The Fermi level of ribbon *r*_*1*_~*r*_4_ is assumed as *E*_*f1*_~*E*_*f4*_. The Fermi level of each curve: S_1_, *E*_*f1*_ = *E*_*f2*_ = *E*_*f3*_ = *E*_*f4*_ = 0.64 eV; S_2_, *E*_*f1*_ = 0.75 eV, *E*_*f2*_ = 0.73 eV, *E*_*f3*_ = 0.71 eV, *E*_*f4*_ = 0.70 eV; S_3_, *E*_*f1*_ = 0.53 eV, *E*_*f2*_ = 0.54 eV, *E*_*f3*_ = 0.54 eV, *E*_*f4*_ = 0.54 eV.

In Fig. 8(a), we investigate the robustness of the proposed broadband absorber under the oblique incidence. It is obvious that the spectral position of the absorption window remains almost constant for angles of incidence from 0° to 15°. Overall, the absorption exceeds 70% for incidence angles varying from 0° to 30°.

To confirm the tenability, we calculate the absorption spectra of normal incidence at different Fermi level, as shown in Fig. 8(b). In order to optimize the absorption spectra, the Fermi level can be adjust independently, as done in double layers absorber. There are three absorption spectra (S_1_, S_2_ and S_3_) in Fig. 8(b). The Fermi level of the three curves are: S_1_, *E*_*f1*_ = *E*_*f2*_ = *E*_*f3*_ = *E*_*f4*_ = 0.64 eV; S_2_, *E*_*f1*_ = 0.75 eV, *E*_*f2*_ = 0.73 eV, *E*_*f3*_ = 0.71 eV, *E*_*f4*_ = 0.70 eV; S_3_, *E*_*f1*_ = 0.53 eV, *E*_*f2*_ = 0.54 eV, *E*_*f3*_ = 0.54 eV, *E*_*f4*_ = 0.54 eV. The bandwidth of S_2_ is 0.8 THz (from 10.7 to 11.5 THz) with the absorptivity exceeds 80%, and the bandwidth of S_3_ is 1.2 THz (from 8.8 to 10.0 THz) with the absorptivity exceeds 90%. As shown, the absorption window shift toward higher frequency (blue-shift) with the increase of Fermi level, and the absorption window shift reversely (red-shift) with the decrease of the Fermi level. Through the research above, the dynamically tenability of the multiple layers broadband absorber is obtained.

## Discussion

In conclusion, tunable broadband absorbers based on multi-layer graphene ribbons has been proposed and investigated. This structure is composed of several graphene/dielectric layers stacking on an optically thick golden substrate, in which the graphene ribbons behave as phase modulators. Because of the phase matching between the regions with and without graphene ribbons, the reflected light is suppressed in a broadband spectrum range. It is significant that the bandwidth of the absorber can be broaden unlimitedly as long as the added graphene layers are properly located in Efficient Absorption Range. Furthermore, the advantage of the graphene-based broadband absorber is that the Fermi level of graphene can be dynamically tuned by applying external gate voltages, without re-fabricating a new structure. By adjusting the Fermi level of the graphene ribbons, the absorption window can be adjusted dynamically. Meanwhile, the presented device exhibits good absorption stability over a wide range of incidence around ±30° at least. Thus, such a broadband absorber provides a novel way for the fabrication of nanophotonic devices for optical absorption in the terahertz region.

## Method

The optical conductivity of graphene in terahertz and infrared region is dominated by the intraband transition, which can be described by a simplified semi-classical Drude model^40^,

1 $$\sigma (\omega )=\frac{{e}^{2}{E}_{f}}{\pi {\hslash }^{2}}\frac{i}{\omega +i{\tau }^{-1}}$$

where *e* is the electron charge, ℏ is the reduced Planck’s constant, *ω* is the angular frequency, *τ* is the electron relaxation time, and *E*_*f*_ is the Fermi level. The electron relaxation time *τ* is calculated from *τ* = *μE*_*f*_/*ev*_*f*_^2^. We take the carrier mobility *μ* = 10^4^ cm^2^/Vs, the Fermi level *E*_*f*_ = 0.64 eV and the Fermi velocity *v*_*f*_ = 10^6^ m/s. The Fermi level of the graphene ribbons is associated with gate voltage. Because of the dependence of conductivity on the Fermi level, the resonance frequency (*f*_*res*_) of the absorber can be adjusted by changing the Fermi level, and has a relation ${f}_{res}\propto \sqrt{{E}_{f}}$ ^41^. In the simulation, we apply transition boundary condition that allocates the conductivity to a single interface with effective thickness *t*_*g*_ = 0.5 nm instead of a film with finite thickness. Such replacement can greatly decrease meshing load and simulation time. The permittivity of graphene can be calculated from *ε*_*g*_ = 1 + *iσ*_*g*_/(*ωε*_0_ *t*_*g*_), where *ε*_0_ is the vacuum permittivity. The permittivity of the dielectric *ε*_*d*_ is 1.90. Furthermore, the Drude model is applied to fit the experimental data of permittivity of gold for the fully reflecting mirror.

### Data availability statement format guidelines

The datasets generated and analysed during the current study are available from the corresponding author on reasonable request.
